# A novel mutation in the OAR domain of *PITX3* associated with congenital posterior subcapsular cataract

**DOI:** 10.1186/s12881-019-0782-2

**Published:** 2019-03-20

**Authors:** Qi Fan, Dan Li, Lei Cai, Xiaodi Qiu, Zhennan Zhao, Jihong Wu, Jin Yang, Yi Lu

**Affiliations:** 1 Department of Ophthalmology and the Eye Institute, Eye and Ear, Nose, and Throat Hospital, Fudan University, 83 Fenyang Rd, Shanghai, People’s Republic of China; 2 The Key Laboratory of Myopia, Ministry of Health, 83 Fenyang Rd, Shanghai, People’s Republic of China; 3 Shanghai Key Laboratory of Visual Impairment and Restoration, 83 Fenyang Rd, Shanghai, People’s Republic of China; 4 Key National Health Committee Key Laboratory of Myopia, Fudan University, 83 Fenyang Rd, Shanghai, People’s Republic of China; 5 Laboratory of Myopia, Chinese Academy of Medical Sciences, 83 Fenyang Rd, Shanghai, People’s Republic of China

**Keywords:** Congenital cataract, *PITX3*, The OAR domain

## Abstract

**Background:**

Congenital cataract is the most common cause of blindness among children worldwide. The aim of this study was to identify causative mutations in a Chinese family with isolated autosomal dominant posterior subcapsular cataract.

**Methods:**

The proband and her parents underwent full ophthalmological examinations. DNA was extracted from the participants’ peripheral venous blood. The mutation was identified via panel-based next-generation sequencing (NGS) and was validated via Sanger sequencing.

**Results:**

Posterior subcapsular lenticular opacity was observed in both of the proband’s eyes. The novel deletion mutation c.797_814del, p.Ser266_Ala271del in the *PITX3* gene was identified in the proband and her father. This mutation is located within the *otp/aristaless/rax* (OAR) domain at the COOH-terminus of the protein, which functions in DNA binding and transactivation. This mutation would result in a deletion of 6 amino acid residues at the C terminal of the protein.

**Conclusions:**

The mutation c.797_814del, p.Ser266_Ala271del is a novel mutation in the conserved DNA-binding OAR domain of *PITX3* that causes congenital cataract.

**Electronic supplementary material:**

The online version of this article (10.1186/s12881-019-0782-2) contains supplementary material, which is available to authorized users.

## Background

Congenital cataract is defined as lens opacity that occurs at birth or during the first decade of life. With an estimated incidence of 1 to 7.2 cases per 10,000 live births [1–3], congenital cataract is responsible for 10 to 38% of pediatric blindness worldwide [4]. In approximately 70% of cases [5], congenital cataract presents as an isolated eye abnormality. Congenital cataract can also occur in association with other ocular and/or systemic abnormalities, such as anterior segment mesenchymal dysgenesis (ASMD), Peters anomaly, Lowe syndrome, and Nance-Horan syndrome.

Between 8.3 and 25% of congenital cataracts are hereditary [6]. To date, genetic studies have identified over 100 causative genes for congenital cataract, including many with minor additional features (e.g., *ABCA3* [7], *CRYAA* [8]). Among these genes, over 30 causative genes have been implicated in association with isolated inherited congenital cataract. These genes may be arbitrarily divided into four major groups [9]: crystallin genes, membrane protein genes, cytoskeletal protein genes, and DNA- or RNA-binding protein genes.

In recent years, the diagnostic use of gene panel next-generation sequencing (NGS) techniques have become commonplace for individuals with hereditary diseases [10]. It has enabled the analysis of a greater number of genes, with the advantage of being fast and inexpensive. We customized a gene capture panel to identify genetic defects in ophthalmic diseases. It was designed to encompass the exons and untranslated (UTR) regions of 790 genes related to eye disease, which included retinal disease genes (*FZD4, NDP, LRP5, TIMP3,TLR4, ABCA4, RP1,* and *RP2*, among others), neuro-ophthalmic disease genes (e.g., *CRX, CRB1, OPA1, OPA3*), corneal disease genes (e.g., *CHST6, TCF4, ZEB1*), glaucoma genes (e.g., *ASB10, OPTN, MYOC*) and others. In particular, this gene panel contains all of the reported causative genes for congenital cataract, including the *CRYAA, CRYAB* and *PITX3* gene.

In this paper, we present two members of a four-generation family with isolated autosomal dominant posterior subcapsular cataract. The aim of this study was to identify causative mutations in the proband and her family that resulted in congenital cataract by using a gene panel.

## Methods

All procedures were performed in accordance with the Declaration of Helsinki and were approved by the Ethics Committee of the Eye & ENT Hospital of Fudan University. Written informed consent was obtained from all individuals who participated in this study and from responsible adults on behalf of minors.

### Patients

Three members of a four-generation Chinese family were enrolled in this study, including the proband (IV:3) and her parents (the father III:5 and the mother III:6). Two of them were affected with autosomal dominant congenital cataract (the proband IV:3 and her father III:5). They received a comprehensive ophthalmological examination at the Department of Ophthalmology of the Eye & ENT Hospital of Fudan University. This examination included tests of uncorrected visual acuity (UCVA) and best corrected visual acuity (BCVA), Goldmann applanation tonometry, and detailed slit-lamp and fundus examinations. Others family members were not enrolled, but their affected status was obtained from the medical records of the local hospital from previous ophthalmological examinations.

### Panel-based next-generation sequencing

Five milliliters of peripheral venous blood from the three participants was collected in tubes containing EDTA. DNA was isolated from the whole-blood samples using a Gentra Puregene Blood Kit (Qiagen, Valencia, CA) in accordance with the manufacturer’s protocol and was stored at − 20 °C until used for sequencing. No DNA from the other family members was available for targeted mutation testing.

All three DNA samples were subjected to analysis using panel-based NGS that involved a targeted gene region capture protocol to identify genetic defects. This protocol and the capture probes were custom designed and produced by Beijing Genomics Institute (BGI, Shenzhen, China), as previously reported. [11] The output sequence data were aligned to the reference human genome UCSC hg38 using Burrows-Wheeler Aligner, version 0.7.10. The variants were filtered to eliminate benign variants with MAF (minor allele frequency) > 0.1% in the 1000 Genomes dataset, and the dbSNP, EXAC, Tumor Heart Freq_Hom, ESP6500, and internal databases. All 790 genes were considered equally. Finally, variant prioritization was performed by combining the total depth, quality score, MAF, potential deleterious effect and the existence of mutation reports in common databases, such as The Human Gene Mutation Database (HGMD), The Retinal Information Network (RetNet), ClinVar, and Online Mendelian Inheritance in Man (OMIM). Variants were classified as benign, likely benign, pathogenic, likely pathogenic or novel variants of uncertain clinical significance according to the American College of Medical Genetics and Genomics (ACMG) guidelines [12].

### *PITX3* gene mutation validation

Sanger sequencing was used to confirm the detected variant. First, polymerase chain reaction (PCR) was performed on the sample using the primers F:5′-GTGTCCTGCCCTTATGCCTC-3′ and R:5′-GGAGGCTGTGAATCGTTGC-3′ for *PITX3*. The PCR program included activation at 94 °C for 3 min; followed by 30 cycles of 94 °C for 20 s, 60 °C for 20 s, and 72 °C for 40 s; and a final extension at 72 °C for 3 min. The PCR products were subjected to direct DNA sequencing by Generay Biotechnology Co., Ltd.

## Results

### Clinical features

**Fig. 1:**
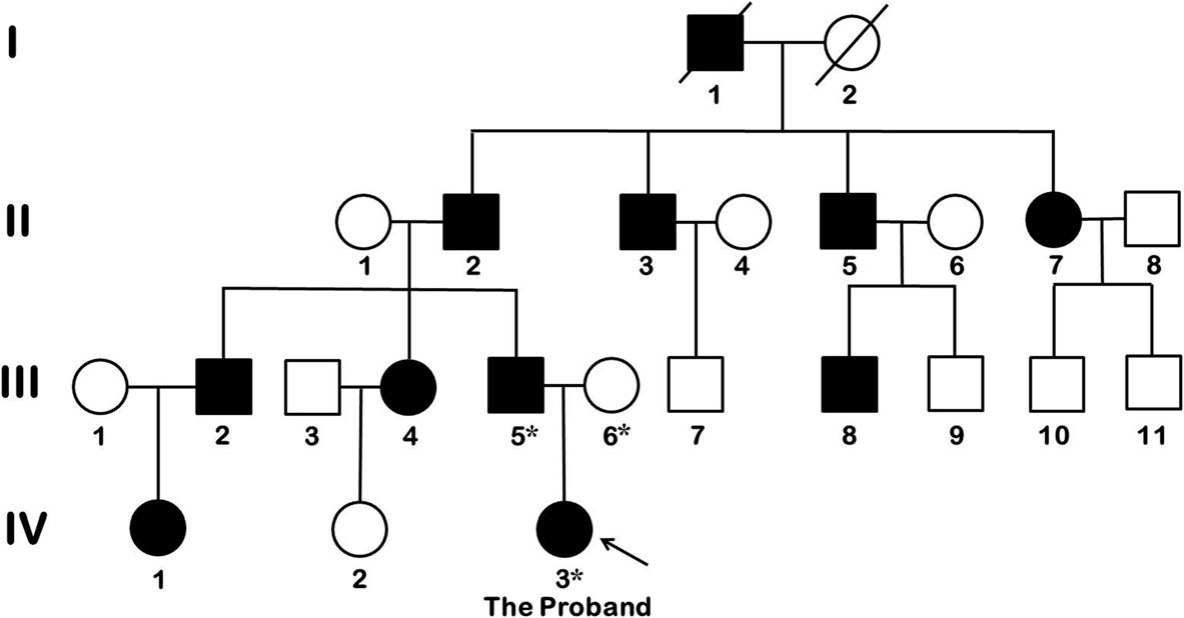
Pedigree of the family enrolled in this study. Squares and circles represent males and females, respectively. Open and solid symbols indicate unaffected and affected individuals, respectively. Astericks indicate enrolled individuals. The proband, IV:3, is indicated with an arrow

**Fig. 2:**
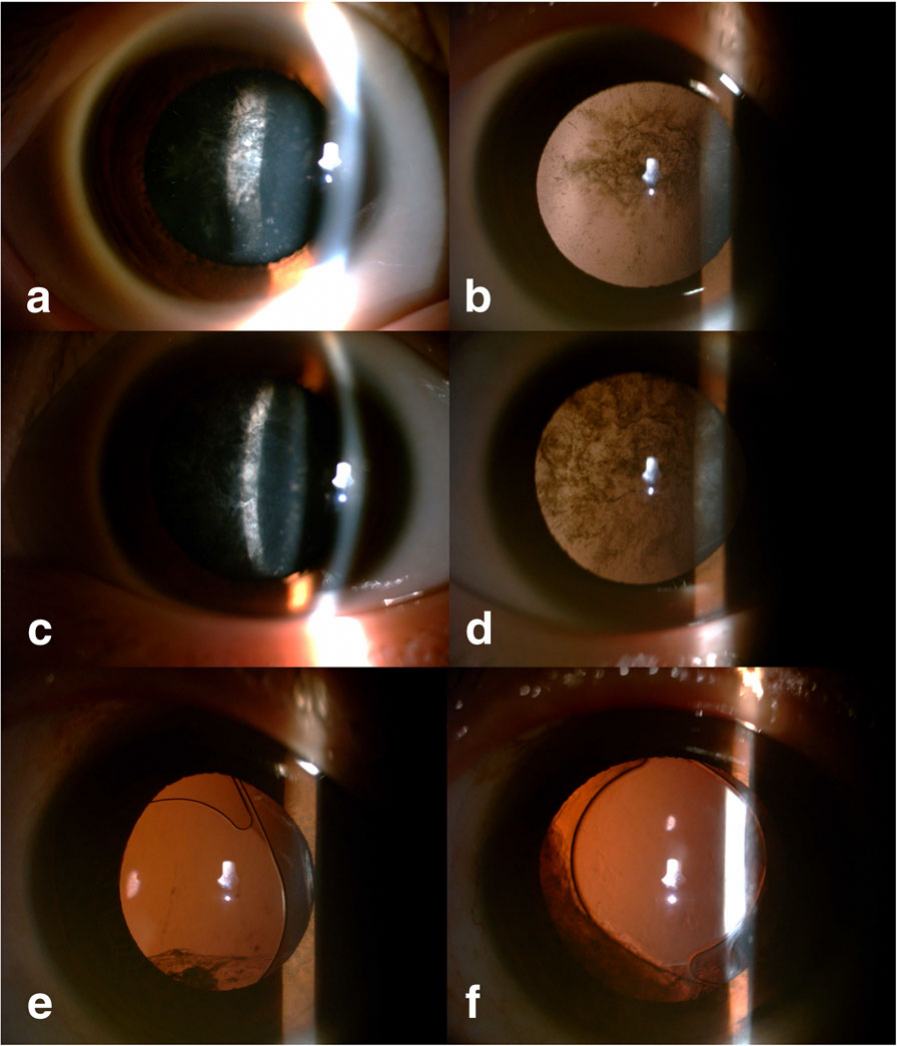
Clinical features of participants. Direct illumination and retroillumination slit-lamp photographs of the proband’s right eye (**a** and **b**) and left eye (**c** and **d**). Posterior subcapsular lenticular opacity was observed in both eyes, and more heavily in the left eye than in the right eye. Retroillumination slit-lamp photographs of the proband’s father’s (III:5) right (**e**) and left (**f**) eye. He underwent cataract extraction with intraocular lens implantation, and pseudophakia in both eyes and mild IOL dislocation in the right eye are apparent

The pedigree of the family enrolled in this study is shown in Fig. 1 (Only the proband and her parents were enrolled in the study). There are four generations that include 11 affected individuals, five unaffected individuals and 8 spouses. The proband (IV:3) was born with bilateral congenital cataract. Her BCVA was 0.4 (logMar unit) in her right eye and 1.0 (logMar unit) in her left eye when she was first referred to our hospital at the age of 10. Posterior subcapsular lenticular opacity was observed in both eyes, with heavier opacity in the left eye than in the right eye (Fig. 2 a, b, c and d). Cataract extraction with posterior capsulotomy, anterior vitrectomy and intraocular lens (IOL) implantation was performed. One year after surgery, both eyes reached a BCVA of 0.1 (logMar unit), with a clear optical axis. The proband’s father (III:5) had also suffered from congenital cataract since childhood. He underwent cataract extraction with IOL implantation at the age of 18, resulting in a bilateral BCVA of 0.7 (logMar unit). He presented with pseudophakia in both eyes and mild IOL dislocation in the right eye (Fig. 2 E and F). The patients had no ocular or systemic abnormalities other than cataract.

### *PITX3* mutation analysis

**Fig. 3:**
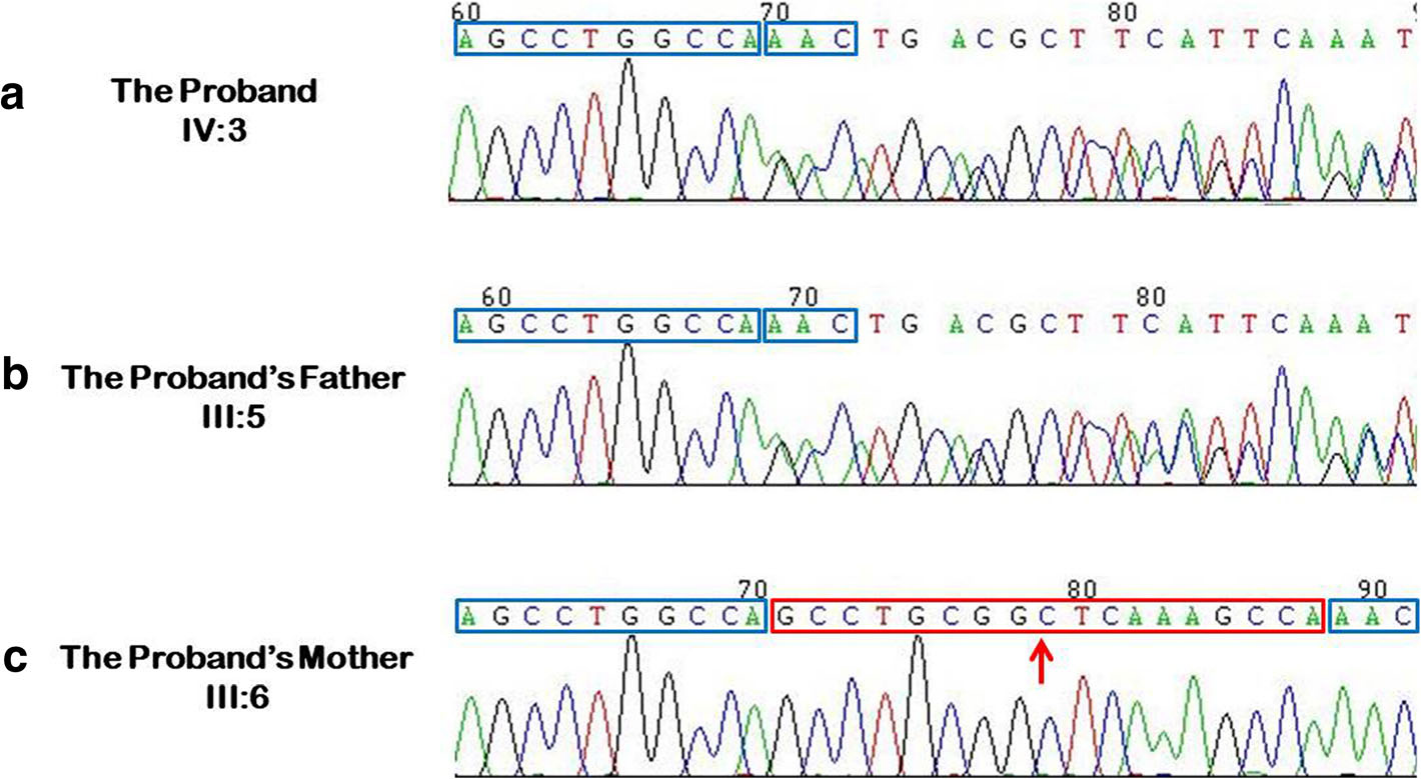
Sequencing analysis of the *PITX3* mutation. Sequence analysis of *PITX3* showing an 18 bp deletion in the affected individuals IV:3 (**a**) and III:5 (**b**). The wild-type sequence is shown in **c**, and the nucleotides deleted in **a** and **b** are indicated using a red box and a red arrow

**Fig. 4:**
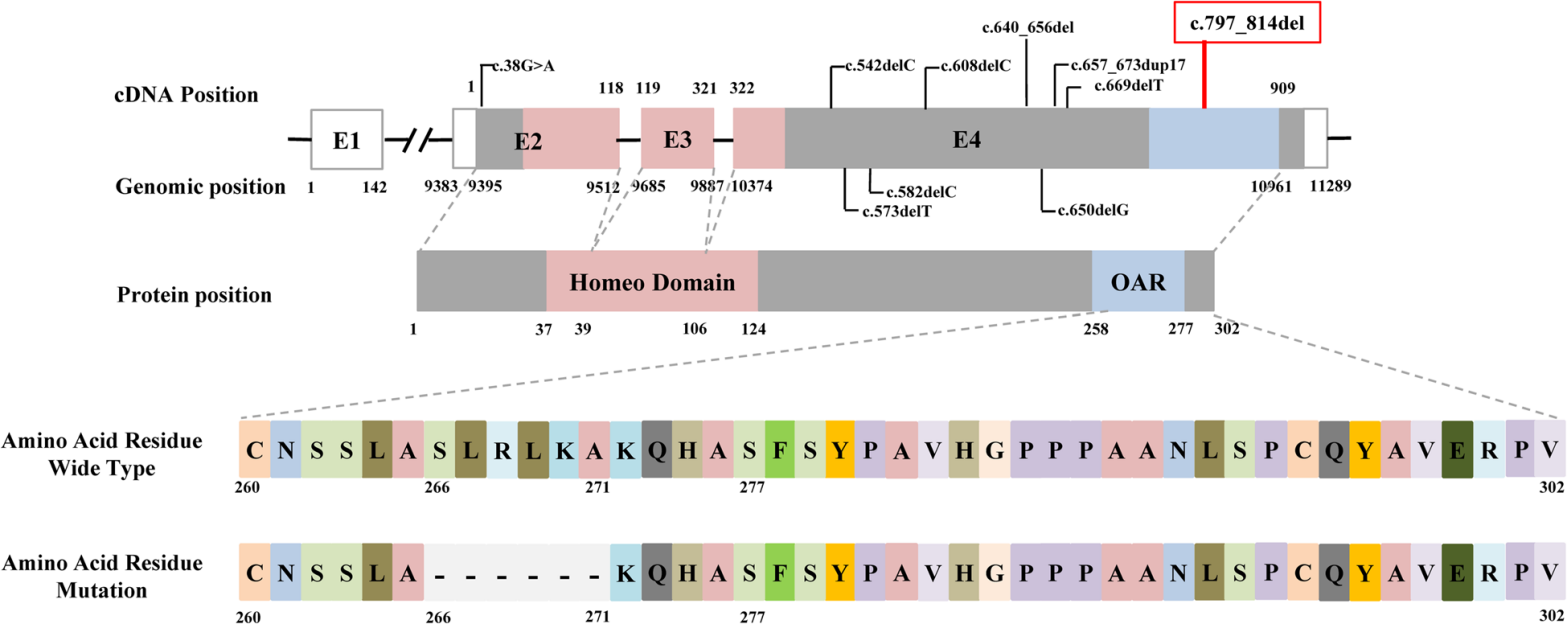
Diagrammatic representation of the PITX3 gene, domains of the PITX3 protein, positions of mutations and amino acid residues. Human *PITX3* consists of four exons (E1-E4) and contains two functional domains: the homeobox domain and the OAR domain. Exons 2 through 4 in *PITX3* encode the PITX3 protein; exons 2 through 4 encode the homeodomain (pink), and exon 4 encodes the OAR domain (blue). Nine reported causal mutations associated with congenital cataract that have been identified in independent studies are indicated in black; none of these mutations is located in the functional domain. The deletion mutation c.797_814del, p.(Ser266_Ala271del) identified in this research, which is indicated using a red box, is located in the OAR domain-encoding region. This mutation will result in a deletion of 6 amino acid residues at codons 266 to 271 at the C-terminus of the protein

After the data acquisition and analysis, a total of 14,076 raw variants were found in the 790 genes, and 211 variants (Additional file 1: Table S1) met the filtering criteria. Based on valuable information in published literatures, we included the genes that were of autosomal dominant genetic inheritance pattern and congenital cataract. Finally, one deletion mutation (c.797_814del, p.Ser266_Ala271del) in *PITX3* was identified as a potentially pathogenic mutation. This mutation was identified in IV:3 and III:5 (Fig. 3) and was validated by Sanger sequencing. The *PITX3* deletion mutation had not been recorded in the public mutation GnomAD database (http://gnomad.broadinstitute.org/). The mutation is classified as an uncertain clinical significance mutation according to the ACMG guidelines (criteria PM2, PM4, and PP1). It is located within the *otp/aristaless/rax* (OAR) domain at the C-terminus of the protein (Fig. 4), which functions in DNA binding mediated by protein-protein interactions. This mutation would result in a deletion of 6 amino acid residues at the C-terminus of the protein (266–271, from a total of 302).

## Discussion

There have been many commercial gene panels used in clinical practice [13]. Gene panels are very helpful in diagnosing hereditary diseases with genetic and phenotypic heterogeneity, including hereditary eye diseases. They have the capacity to identify a large number (from tens to hundreds) of genes with potential clinical utility. They can provide better sensitivity for point mutations, deletion/insertion mutations within 20 bp and homozygous deletions in exons. However, a limitation of gene panel assays is that they are not capable of large copy number variation and genomic structural variation. Additionally, they not able to explore possible causative mutations outside the gene list contained in the panel.

In this study, we detected the c.797_814del, p.Ser266_Ala271del mutation, a novel deletion variant of *PITX3* that is associated with congenital posterior subcapsular cataract, by using a genetic testing panel. *PITX3* is a DNA-binding protein gene, which are often associated with more complex phenotypes such as ASMD. However, the family described in this paper does not appear to have any additional features.

*PITX3*, which belongs to the PITX/RIEG family of homeobox genes, contains characteristic and strongly conserved homeobox and C-terminal OAR domains that function in DNA binding and transactivation. It plays a critical role in normal lens development during vertebrate eye formation. Mutations in *PITX3* are linked to developmental abnormalities of the anterior eye, particularly the lens. Knockdown of Pitx3 in mouse induced small eyes without lenses [14]. In addition, mutations in *PITX3* are responsible for various ocular defects [15], including congenital cataract, ASMD, Peters anomaly, and microphthalmia.

*PITX3* mutations play a role in the pathogenesis of congenital cataract, including both dominant and recessive types of cataract with or without other ocular abnormalities. Previously, 9 unique *PITX3* mutations [16, 17] (c.38G > A, p.Ser13Asn; c.542delC, p.Pro181LeufsX127; c.573delC, p.Ser192AlafsX117; c.582delC, p.Ile194MetfsX115; c.608delC, p.Ala203GlyfsX105; c.640_656del, p.Ala214ArgfsX42; c.650delG, p.Gly217AlafsX91; c.669delC, p.Leu225TrpfsX84; and c. 640_656dup17, p.Gly220ProfsX95) that cause congenital cataract have been reported. Except c.38G > A, p.Ser13Asn mutation, other mutations are in exon 4 and are frameshift mutations. The c.640_656del, p.Ala214ArgfsX42 mutation is recessive, while the rest are dominant. The 17 bp duplication mutation c. 640_656dup17, p.Gly220ProfsX95 has been identified as the most common of these mutations in humans since it was first described by Semina et al. [18]. However, it is interesting that no mutation had previously been identified in either the homeodomain- or the OAR domain-encoding region of *PITX3*. The mutation identified in this paper is a mutation located within the OAR domain-encoding region.

The OAR domain is a strongly conserved functional domain. Previous studies have revealed that it is involved in specific protein-protein interactions and plays multiple regulatory roles. It functions as an intramolecular switch of transactivation activity in the Cart1 homeoprotein by inhibiting DNA binding [19]. It also serves as a repressor of transcription in *PITX3*; loss of the OAR domain leads to an enhancement of *PITX3* transcription and translation [20]. Simultaneously, it is essential for ensuring the normal expression of downstream genes of *PITX3*, such as Mip and Foxe3, via protein-protein interactions [20]. Although the functions of the OAR domain are not yet well understood, it has been proven that disruption of the OAR domain of the *PITX3* protein is associated with alterations in DNA-binding profiles and/or transactivation activities [21].

The deletion mutation in this study was an in-frame deletion mutation. It resulted in a deletion of 6 amino acid residues at the C-terminus that altered the OAR domain. Interestingly, the majority of previously reported-*PITX3* mutations were out-of-frame deletions that lead to the disruption of the OAR domain, while this in-frame deletion would not be expected to do so. Perhaps its DNA-binding profile and/or transactivation activities may have been altered. However, whether the OAR domain folding or the DNA-binding profile and/or transactivation activities were altered was not clear. Further functional analyses and DNA-protein binding assays are required to confirm the molecular mechanism.

## Conclusions

This investigation reports a novel mutation in *PITX3*, which adds to our knowledge of the mutations in *PITX3* that cause inherited cataract. The majority of reported mutations in *PITX3* result in a loss of the OAR domain, as they cause frameshifts prior to the OAR domain, while the mutation identified in this paper was located within the OAR domain-encoding region. However, it is a limitation of this study that more distant family members could not be recruited and tested. The exact pathogenicity of the mutation is still unclear; therefore, further experiments are needed to investigate the mechanisms of mutation in the *PITX3* pathway during lens development.

## Additional file

Additional file 1: **Table S1.** The full filtering variants of the genetic testing panel. (XLSX 62 kb)

## Abbreviations

BCVA: Best corrected visual acuity
IOL: Intraocular lens
NGS: Next-generation sequencing
OAR domains: *Otp/aristaless/rax* domains
PCR: Polymerase chain reaction
*PITX3*: Paired-like homeodomain 3 transcription factor
UCVA: Uncorrected visual acuity
UTRs: Untranslated regions
